# Agro-Food Waste as an Ingredient in Functional Beverage Processing: Sources, Functionality, Market and Regulation

**DOI:** 10.3390/foods12081583

**Published:** 2023-04-08

**Authors:** Xóchitl Alejandra Pérez-Marroquín, Ana Guadalupe Estrada-Fernández, Adelfo García-Ceja, Gabriel Aguirre-Álvarez, Arely León-López

**Affiliations:** 1 Instituto de Ciencias Agropecuarias, Universidad Autónoma del Estado de Hidalgo, Av. Universidad Km. 1 Rancho Universitario, Tulancingo C.P. 43600, Hidalgo, Mexico; xochitl_perez@uaeh.edu.mx (X.A.P.-M.); aguirre@uaeh.edu.mx (G.A.-Á.); 2 Instituto Tecnológico Superior del Oriente del Estado de Hidalgo, Carretera Apan-Tepeapulco Km 3.5, Colonia Las Peñitas, Apan C.P. 43900, Hidalgo, Mexico; aestrada@itesa.edu.mx; 3 Instituto Tecnológico Superior de Venustiano Carranza, Av. Tecnológico S/N, Col. el Huasteco, Ciudad Lázaro Cárdenas, Venustiano Carranza C.P 73049, Puebla, Mexico; adelfo.ceja@vcarranza.tecnm.mx

**Keywords:** food waste, agro-food industry, functional beverages, regulation

## Abstract

Waste generated from the agro-food industry represents a concerning environmental, social and economic issue. The Food and Agriculture Organization of the United Nations defines food waste as all food that decreases in quantity or quality to the extent that it is thrown out by food service providers and consumers. The FAO reports that 17% of worldwide food production may be wasted. Food waste may include fresh products, food close to the expiration date discarded by retailers and food products from household kitchens and eating establishments. However, food waste offers different possibilities to extract functional ingredients from different sources, such as dairy, cereals, fruits, vegetables, fibers, oils, dye and bioactive compounds. The optimization of agro-food waste as an ingredient will help in the development and innovation of food products to generate functional food and beverages to prevent and treat several diseases in consumers.

## 1. Introduction

**Figure 1:**
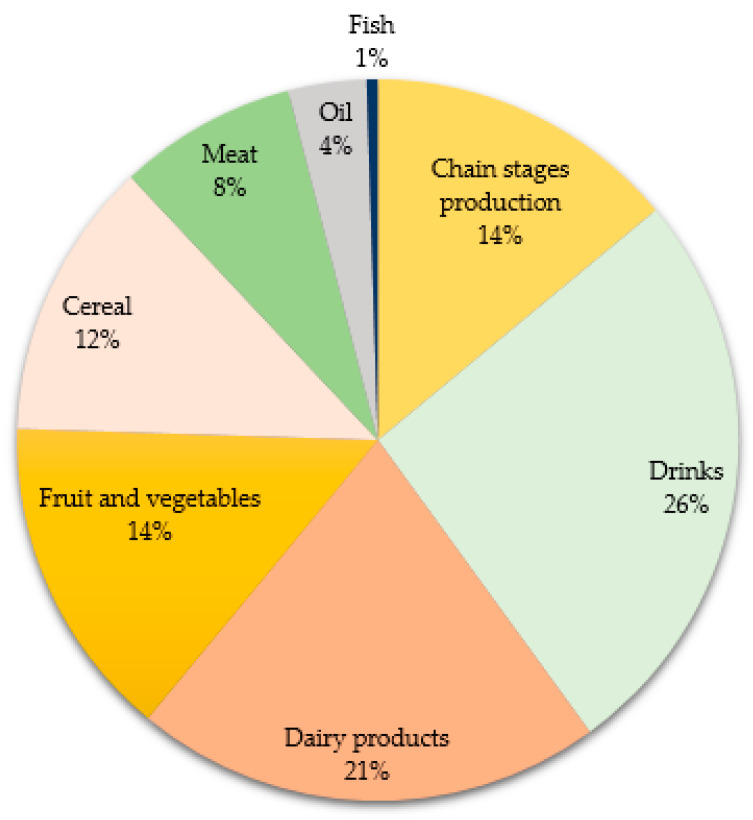
Production of agro-food waste in different industries [7,9].

At present, waste from the agro-food industry is now both an environmental problem and a major social and economic issue. The Food and Agriculture Organization of the United Nations (FAO) refers to food waste as all food that is compromised in quantity or quality by food providers and consumers. Food waste comes in many forms: (a) fresh products that are removed from the supply chain during sorting operations and do not have the optimal size, color and/or other characteristics of the base product; (b) food that is close to its expiry date and is discarded by retailers and consumers; and (c) all food from household kitchens and food service operations [1,2,3,4]. Food waste can also be food that has lost quantity, quality and/or safety at any stage of the food processing chain, including during post-production processes such as handling, storage, transport and processing, preservation and packaging [1,3]. Food waste mainly includes vegetable waste such as peels, stems, seeds, shells, bran, pulp and residues. Animal sources include waste from animal husbandry, dairy processing, seafood and the slaughter process [5,6]. The FAO reports that 17.0% of global food production is generated as waste and 14.0% of food production is lost during the production chain. A total of 26.0% of food waste comes from the beverage industry, 21.0% from the dairy industry, 14.5% from fruit/vegetable production and processing, 12.5% from grain processing, 8.0% from meat processing and preservation, 3.5% from the animal and vegetable oil industries and 0.5% from fish production and processing (Figure 1) [7,8,9]. Food waste is usually disposed of in landfills or used to make compost, but the waste is a potential source of value-added compounds (phytochemicals, antioxidants, color pigments and nutrients) that have some nutritional value and functional properties [10,11]. Nowadays, the proper use of this type of waste as raw materials or food additives offers the beverage industry many opportunities for innovation, enabling the generation of products with better nutritional and sensory characteristics, including higher protein, fiber and mineral contents, and desirable qualities such as antioxidant, microbial and antihypertensive properties, helping to enhance human health [12,13,14]. This research provides an overview of agro-food waste from fruits and vegetables, dairy products, cereals and fish, emphasizing their value as ingredients in the production of functional beverages and their market and regulation.

## 2. Functional Food and Beverages

Food is essential to survival, yet food products often fail to perform their primary function, which is feeding consumers [15]. Today, the food industry faces a wide range of challenges related to social issues such as climate change, environmental protection, commercial margins, legislation, food security and the growing demand for food [16,17]. As a result of these issues, the environment, health, economy and food manufacturing have changed from classic engineering, where the food products were made based on technology and raw materials, placing the consumer’s needs at the end of the food chain, to a modern design based on reverse engineering [18,19]. The main objective of the food industry is to satisfy the preferences, interests and needs of consumers [20]. In recent decades, consumers have demanded food products with nutritional characteristics and also products to prevent and treat certain diseases. Consequently, functional foods began to be developed in 1980 in Japan [21,22,23].

Functional foods are foods that contain bioactive ingredients that provide benefits other than nutritional effects. They contribute to some human functions, preventing and/or mitigating the development of chronic diseases (hypertension, obesity, cancer, diabetes and others). However, functional foods are regulated but not legally recognized in most countries [21,22,23,24,25]. Functional foods exist in a wide range of forms including bakery products, baby foods, cereal-based foods, dairy products, meat products and beverages [26,27]. Functional beverages are the most important segment of functional food products due to three important factors: (a) easier storage of products during refrigeration time; (b) higher availability to incorporate bioactive components and nutrients; and (c) greater satisfaction of consumer demand in terms of appearance, size, shape and content of the container [28,29]. Another factor influencing the growth of the functional beverage market is the consumer interest in natural and organic ingredients [21]. This trend offers opportunities in the food industry, and especially the functional beverage sector, to develop new formulations and preparations of beverages from natural sources such as cereals, dairy products, herbs, etc. [28,30,31].

The extraction of different compounds from food waste generates new options to utilize these in the food industry for the development of food products with better characteristics [12]. Different innovative technologies exist to extract functional compounds from food waste [32]. The advantage of these technologies is that they present fewer environmental problems because they do not require a high volume of solvents. The most-used technologies for extraction are: ultrasound (UAE), microwave (MAE), pressurized liquid extraction (PLE) and enzyme-assisted extraction (EAE).

Ultrasound-assisted extraction (UAE): Ultrasound waves cause disruption in the plant tissue through physical forces developed during acoustic cavitation, helping to release extractable components in less time. Ultrasound extracts bioactive compounds from fruit and vegetable waste and food by-products [33,34,35].

Microwave-assisted extraction (MAE): This technology involves the use of microwave radiation energy to heat up the solute–solvent mixture. The diffusion of the solvent through the sample increases the disruption of hydrogen bonds holding the sample, thereby allowing the target compounds to dissolve into the extraction fluid [33,36].

Pressurized liquid extraction (PLE): This is one of the current techniques in development for extracting phytochemicals because it requires the use of a high temperature (40–200 °C) and pressure (3.3–20.3 MPa). This facilitates the desorption and solubility of molecules [37,38].

Enzyme-assisted extraction (EAE): This technique exploits the capability of enzymes to break down the cell wall compartment, ensuring the movement of the cytoplasmatic content in extraction fluid such as water. This method has been identified as an eco-friendly method owing to its use of water as a solvent rather than organic solvents [33,39].

## 3. Functional Beverages: Formulation and Classification

One of the main objectives for the development of new beverage formulations is the incorporation of traditional and functional ingredients to enhance the appearance and storage stability to ensure stable sensory characteristics during shelf life [40,41]. The principal ingredients used in beverages are presented in Table 1.

Functional foods and beverages have become an important part of consumer lifestyles [28]. Functional beverages meet the needs of different lifestyles, ages and genders and can also provide benefits, including compensation for a lack of a healthy diet, prevention against specific diseases, fatigue and stress relief, anti-aging properties and an energy boost [43,44]. In recent years, the functional beverage market has developed a growing number of products that stand out for their characteristics, such as improving intestinal or cardiovascular health and aiding the immune system, weight control and anti-aging processes [45]. The main classification of functional beverages presents three categories:

I. Milk beverages: They include probiotics and mineral-fortified drinks. They contain several components, such as peptides, oligosaccharides, enzymes, vitamins and minerals. These components can be beneficial for the health of consumers [46].

II. Fruit and vegetable drinks: The juice industry is the major generator of vegetable waste and fruit beverages. Some of their principal properties are due to the bioactive potential of anthocyanins, flavonoids, epicatechins, flavonones, procyanidins, lignans, carotenoids, soluble and insoluble fiber, isothiocyanates, phenolic acids and vitamins A, C and E. There is the possibility of converting these matrices into functional and personalized products with health benefits [31].

III. Sports drinks: Play an important role in hydration (improving sports performance) and in the prevention or improvement in specific health conditions. Their formulas can be specifically designed to increase energy, improve mental focus and/or prevent bone and joint pain. The main function of this group of beverages is to hydrate athletes and restore electrolytes, carbohydrates and other nutrients that may be depleted during exercise [29].

### Market and Regulation of Functional Beverages

The functional food and beverage market has grown over the past decade because consumers are increasingly seeking out diets to help prevent illnesses such as osteoporosis and cardiovascular and neurodegenerative diseases, or even to optimize their health by increasing their energy and boosting their immune systems. Additionally, price, taste, health claims, packaging and branding, as well as sensory attributes, have an influence on consumer preferences for functional food and beverages [47,48,49]. The global functional food market was valued at USD 175 billion in 2020 and is predicted to exceed USD 309 billion by 2027. The functional beverage market was valued at USD 110,148.9 million in 2020 and is estimated to reach USD 200,080.3 million by 2030 [50]. This fact has led to the market of functional foods and beverages growing by approximately 10% per year, generating USD 192 billion in 2020 [44,51]. Globally, Japan is the largest functional food market, where 1700 functional foods have been launched. Although, the US and European markets appear to be less developed, these three markets account for 90% of the total sales [52,53]. European countries, including Germany, France, Italy, Hungary, Poland and Russia, represent the most important market for functional food and beverage products. It is a heterogeneous market due to the large cultural differences among customers [54,55,56]. A high percentage of the population in northern European countries such as Finland, Sweden and the Netherlands consumes functional foods; Belgium represents the lowest functional-food consumer percentage [53]. The United States of America leads the functional food and beverage market with an 8.7% annual growth rate [54,57]. However, in Latin America, in countries such as Brazil and Mexico, functional food and beverage development, distribution and trade are relatively new but present a promising rate of growth that is expected to continue [56,58]. Growth in the functional food and beverage market is slow in Australia, New Zealand (NZ), Africa, the Middle East and Eastern Europe due to extreme poverty and socio-cultural factors, representing just 8% of the total revenue worldwide [56,59]. Asia has the largest functional food market. China, Japan and Taiwan house the largest number of health food and beverage manufacturing global enterprises because their citizens are more concerned about health and believe that functional foods are a great way to take care of the body [59,60]. The main market trends that have been driving activity and growth in the functional food and beverage market are:

- Marketplace convergence of a range of categories;
- Accelerated growth in the number of functional foods and beverages;
- Cobranding partnerships between ingredient suppliers and manufacturers;
- Increased focus on science and validation of claims;
- Growing demand for sustainable and environmentally friendly food;
- Expansion of active nutrition products as consumers become more health-conscious;
- The emergence and popularity of innovative dosage and delivery forms;
- The rise of the millennials and a new paradigm on health and wellness [44,61,62].

In general terms, the regulation of functional food and beverage products is still a progressive topic due to the regulation of food supplements, dietetic food and genetically modified organisms (GMO). Japan was the first country to recognize the health benefits of functional foods and the need for the marketing and regulation of these types of foods. In Japan, functional food products are regulated by Food for Specified Health Uses (FOSHU), which requires these types of products to contain an ingredient with health functions and official approval to claim to have physiological effects on the human body [63]. This regulation has influenced other Asian countries such as China, the Republic of Korea and many others to develop and implement regulations for the production and sale of functional foods and beverages [64]. In the European Union, the European Food Safety Authority (EFSA) considers functional foods a concept rather than a specific food category [28,58]. Additionally, the procedure for the validation of health claims is costly and lengthy because of the involved scientific data to support the benefit of a functional products. Therefore, it is difficult to market a product with health claims in European countries because it involves the authorization procedure of every country’s national authority [28]. In Europe, to obtain the authorization to market a functional beverage, a rigorous assessment of the toxicological, nutritional, compositional and other relevant data is needed [65]. In the USA, the Food and Drug Administration (FDA) is responsible for the regulation of functional foods and beverages. They categorize the products into a specific category, underlining specific health benefits [66]. In Brazil, the functional food and beverage regulation has been defined by the Agencia Nacional de Vigilancia Sanitaria since 1999; in Mexico, by the Ley General de Salud since 2011; and in Chile, by the Manual of “Selección de Alimentos” since 1999 [67].

Other countries follow the Codex Alimentarius as a guide to establish the regulation of functional food products. However, every country has its own legislation for functional foods and beverages. [50,68]. The global functional food regulation is determined by the consumers’ acknowledgment of the connection between health and diet [28,69].

## 4. Food Waste as an Ingredient for the Preparation of Functional Beverages

### 4.1. Fruit and Vegetable Waste

**Figure 2:**
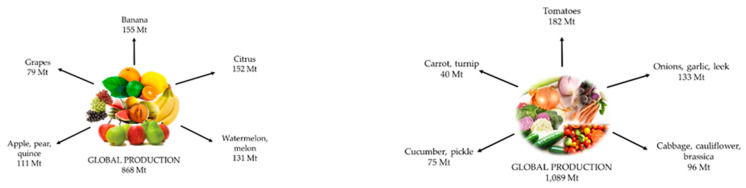
Global production of fruits and vegetables.

Fruits and vegetables are edible parts of plants. They are essential components of the human diet because they are a great source of vitamins, minerals and fiber [70,71]. The FAO (2018) estimates that the global production of fruits and vegetables is currently 868 Mt and 1089 Mt, respectively. However, their production has been increasing due to population growth and changes in dietary habits. Fruit production is led by bananas, citrus, melons, apples, pears and grapes, and the most produced vegetables are tomatoes, onions, garlic, cabbages, cauliflower, brassicas, cucumbers, pickles, carrots and turnips (Figure 2) [71,72,73].

Unfortunately, half of the worldwide fruit and vegetable production ends up as waste, generating environmental problems because these materials are naturally susceptible to microbiological degradation [72,74]. The juice industry is the major generator of vegetable waste [75]. Fruit and vegetable waste (FVW) comprises the inedible parts of food, i.e., the outer layers and extremities of fruits and vegetables that are removed during processing, mainly by peeling and pressing. These parts are discarded during collection, handling, transportation, and processing [75,76]. Utilizing fruit and vegetable waste represents a great opportunity to obtain vitamins, minerals, fiber, oils, dye and bioactive compounds that could be included in the human diet [6,77]. However, its valorization for the recovery of valuable components is limited. In this sense, industry and the research community have been working together to transform fruit waste into high-value-added products [78]. There are some research studies where fruit waste was used as an ingredient in the production of different beverages, showing its important functionality (Table 2) [79,80,81,82,83]. Some of the functionalities in beverages produced from FVW are related to the presence of bioactive compounds such as polyphenols present in fruit peel [84,85,86,87,88,89], seeds [87,89,90] or the final product of fruit processing [91,92,93,94]. In vegetables, the pomace and peel are the main sources of bioactive compounds [74,95,96,97,98,99,100]. They result in excellent antioxidant activity, by radical inhibitions such as DPPH (2,2-Diphenyl-1-picrylhydrazyl), ABTS (2,2′-azino-bis(3-ethylbenzothiazoline-6-sulfonic acid)), and anti-inflammatory activity [101]. Additionally, the antimicrobial activity is related to the presence of bioactive compounds in FVW. This activity increases during the storage of beverages, maintaining their physiochemical (lower sedimentation and better viscosity and color) and nutritional properties (high concentration of vitamins and minerals). FVW is rich in fiber, which helps to increase satiety, control the glycemic index, reduce the risk of heart diseases, alleviate constipation and reduce the risk of metabolic syndromes and diabetes. Moreover, it can increase the viscosity and stability of tea, juice and refreshing and other types of beverages [102,103,104,105,106,107].

### 4.2. Dairy Waste

**Figure 3:**
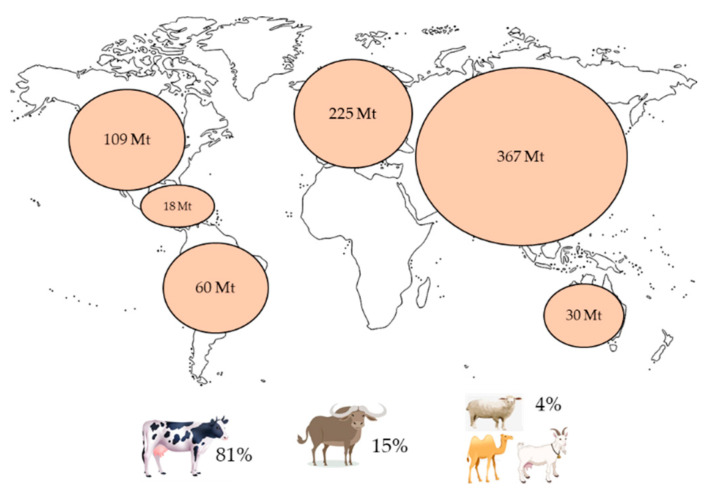
Global milk production and principal types of milk consumed and processed around the world [109].

In 2019, world milk production was estimated to be about 852 Mt, and in 2020, the production was 906 Mt. This production is expected to grow 1.7% per year, reaching 1020 Mt by 2030 (FAO 2021). For 2020, the regions with higher milk production were Asia (367 Mt), Europe (225 Mt) and North America (109 Mt), followed by South America, Oceania, Central America and the Caribbean (Figure 3). The principal types of milk produced globally are cow (81.0%), buffalo (15%) and goat, sheep and camel (4.0% combined) [108,109].

Milk should be processed almost immediately after milking, and it can only be stored for a few days [108]. The majority of dairy products are consumed in fresh, unprocessed or slightly processed (pasteurized or fermented) forms. The main processed dairy products produced around the world are butter (9.3 Mt), cheese (1.8 Mt), whole milk powder (2.7 Mt) and skim milk powder (2.5 Mt). The waste derived from dairy products is around 29 Mt per year, generating an important environmental problem [69,110]. Dairy waste can be classified as wastewater or solid waste [111].

Solid waste: The dairy industry produces around 200–350 kg of sludge for every 500,000 L of milk processed. Sludge contains degradable organic and non-biodegradable solid matter. The amount of sludge produced increases with an increase in wastewater [69,112].

Wastewater: The dairy industry produces 1–3 L of wastewater for every liter of milk produced. It contains a high concentration of organic components such as carbohydrates (lactose), protein, minerals and fats [110,113].

Milk whey is the main by-product of the dairy industry, produced during cheese and casein manufacturing; for every 10 L of milk, 1 kg of cheese and 9 L of milk whey are generated [108]. Milk whey represents an important pollution problem because of its biochemical oxygen demand (BOD) and chemical oxygen demand (COD) in the range of 1–10 g/L and 0.3–5.9 g/L, respectively [69,114]. Whey is a yellowish-to-greenish clear liquid obtained after milk coagulation during the cheese-making process. Whey represents about 85–95% of the milk volume and contains over 55% of milk nutrients such as minerals, proteins and lactose. Although milk whey is considered a waste product, the literature supports that milk whey has relevant nutritional and functional properties that make it suitable for use in functional foods [115,116,117,118].

Table 3 presents fermented beverages produced by dairy waste, mainly whey obtained from different types of cheese production such as Oaxaca, Ricotta and Chhena. Whey can be used in liquid form [26,119,120] or as a functional ingredient in powder form [121,122,123]. Whey beverages show functional activities such as antioxidant, antibacterial and cytomodulatory properties and also inhibit angiotensin-converting enzymes (ACE). The nutritional value of beverages produced using whey is higher; hence, they have good acceptance among consumers [124,125,126,127].

### 4.3. Cereal Waste

**Figure 4:**
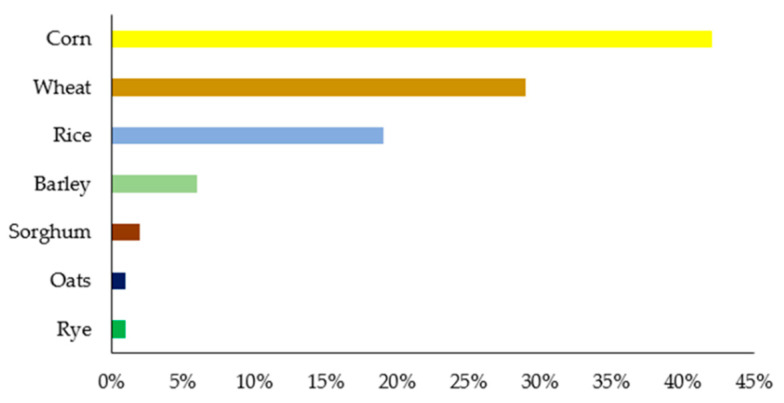
Global cereal production 2020–2021 [4].

Cereal consumption provides more than 56% of human energy due to its carbohydrates, especially starch, and makes up 50% of the protein consumed worldwide [128]. Cereals are also rich in protein, vitamins and fiber. They also contain a small proportion of minerals (K, P, Mg, Ca and Fe) and unsaturated fatty acids [129,130,131]. The main cereals produced and consumed in the human diet are corn, wheat, rice, barley, oats, sorghum and rye (Figure 4). Cereal processing is one of the most important industries in the agro-food sector because cereal food products cover over 20% of the daily diet [132,133]. Cereal products are obtained by dry milling (wheat and rye), pearling (rice, oat, barley), wet milling (corn, wheat) and malting (barley, corn, wheat). This processing can generate solid waste (corn pericarp, corn grits, brewer’s spent grain, lignocellulosic biomass and baking industry waste) and liquid waste (milling waste water, parboiled rice effluent, corn steep liquor, bakery and tortilla wastewater) [134,135]. Lignocellulosic biomass is particularly suitable as a low-cost carbon substrate for solid-state fermentation [133]. The composition of waste generated in the cereal industry depends on the raw material processed and its operating conditions [110,133,134]. Cereal waste can be used as a low-cost material to extract value-added compounds that have potential health benefits (antioxidant, anti-inflammatory, regulation of hormones, enhancement of the immune system), for example, polyphenols used in nutraceuticals, dietary supplements and functional food formulations [130,133,136].

The main objective of waste management in the cereal industry is to improve resource efficiency while protecting the environment. The techniques used for waste treatment modify their physical, chemical or biological characteristics to reduce their toxicity and/or volume and make the waste safer for disposal [135,137,138].

Today, many by-products derived from cereal processing have reached the market as dietary supplements or ingredients in fermented functional beverages [139]. Fermentation is a cheap biotechnological process used worldwide and is also one of the oldest processes used for food preservation and the elaboration of food products as beverages from various cereals worldwide [140,141,142]. This process uses enzymes and microorganisms (lactic acid bacteria, yeasts and molds) to trigger acidification, proteolysis and/or amino acid conversions to obtain products with desirable characteristics linked to texture, taste and odor and to extend the shelf life of beverages [143,144]. Fermented beverages are an optimum vehicle to transport nutrients (dietary fiber, vitamins, fatty acids, probiotics and minerals) and bioactive compounds (phytochemicals, phytoestrogens, phenolic compounds, flavonoids, carotenoids, etc.) into the body [141,142,145]. Fermented functional beverages are suitable for consumption by vegetarians, vegans and lactose-intolerant consumers. Additionally, they have an economic impact on poor diets and the potential to reduce adverse health effects by acting as antioxidant and anti-inflammatory agents, regulating hormones, enhancing the immune system, etc. [25,141,146]. Beverages produced from cereal waste from sorghum, barley, corn, barnyard millet, oats, wheat, rice, rye or quinoa are often high in soluble fiber, which helps to reduce the glycemic index by slowing down digestion and absorption [147]. Phenolic compounds have antioxidant potential and scavenge harmful free radicals in the body, reducing oxidative stress [148,149,150,151,152]. They also show antihypertensive, nutritional and physiochemical properties that promote physicochemical and functional properties [152,153,154,155,156] (Table 4).

### 4.4. Fish Processing Waste

**Figure 5:**
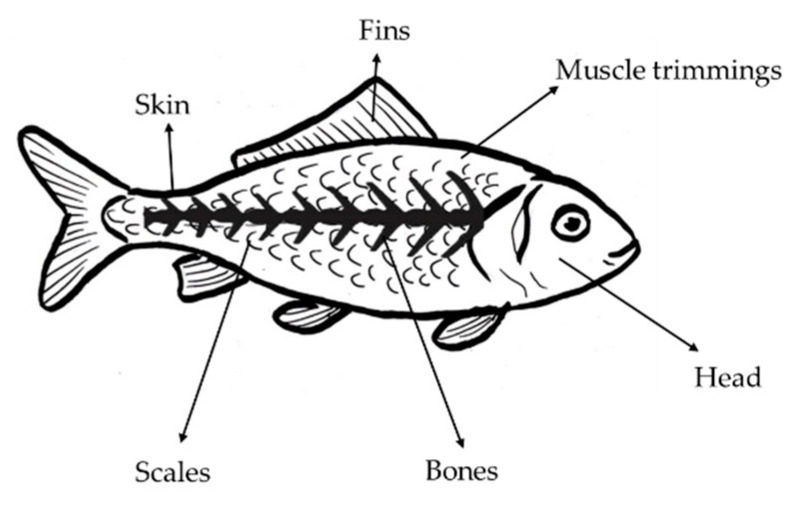
Fish parts that are considered waste [158,160,161].

Almost 214 Mt of fish was processed in 2020, with its production expected to decrease to 202 Mt by 2030 [157]. Around 27% of the total catch of fish is lost or unutilized for consumption due to spoilage or deficient management and storage problems [158,159]. Around 65% of the total fish production is converted into waste. The term “fish waste” (FW) refers to the whole fish when it is damaged. It includes heads (9–12%), bones (9–15%), viscera (12–18%), muscle trimmings (15–20%), skin and fins (1–3%) and scales [160,161] (Figure 5). Traditionally, FW is used in animal feed and fertilizer or is discarded in landfills, causing environmental problems, damage to the marine ecosystem and the generation of unpleasant odors [162,163,164]. FW contains approximately 58% protein, 19% fat and minerals. Additionally, approximately 22% of its content is made up of the fatty acids palmitic acid and oleic acid. FW is an important source of by-products (proteins and amino acids, collagen and gelatin, oil and several enzymes) used in several fields and the food industry a functional ingredients [164,165,166,167]. Some studies have demonstrated the use of by-products obtained from FW in beverages with functional properties such as antioxidants against superoxide, ABTS and DPPH radicals and antimicrobial activity [168,169,170]. It possesses great physicochemical and nutritional properties [165,168,171,172,173]. Functional beverages with FW (gelatin and collagen) have been demonstrated to help increase skin hydration, brightness and texture and to decrease the appearance of crow’s feet wrinkles, pores and spots [174,175,176]. (Table 5).

## 5. Conclusions

Food waste represents an important economic and pollution problem due to the high volume that is generated every day at home and in food industries. Therefore, the conversion of food waste into raw materials that can be used in the food production chain allows the mitigation of this problem, giving added value to what is considered waste. To minimize agro-food waste, some options can be considered, such as reduction, recovery and recycling. The first depends on a supply chain where products with imperfections are left at the place of harvest following marketing standards, considering that the market demands a quality product. Regarding recovery, models for the redistribution of food surpluses could be developed; good logistics can improve the tracking and delivery of food through the different redistribution channels available. Assuming that the two previous proposals are unavoidable, the viable option could be recycling, in which agro-food wastes are used in animal feed, composting or raw material used in renewable energy, contributing to a reduction in pollution. These options may benefit from new approaches to waste valorization that not only consider the extraction of high-value compounds but also the development of products that improve ecosystem services, especially crop production.

Agro-food waste is a viable source for obtaining active ingredients for the preparation of functional beverages. The interest in functional foods in recent years is a result of improved quality of life (providing benefits such as disease prevention, relief from fatigue and stress and an energetic contribution) and the increasing costs of health care, which have encouraged the food industry to develop more effective functional foods and beverages, taking advantage of products generated from food waste. The increased demand for innovative beverages such as functional beverages has been generated due to the prevalence of vegetarianism, veganism and lactose intolerance and consumer interest in being aware of the beneficial effects of food products on the market. However, the regulation of functional beverages and foods is not fully established globally; it is specific to every single country. In Asian and European countries, functional foods have established regulations, while in developing countries, the regulation of functional foods and beverages is limited. Legislation is needed regarding the production and trade of functional foods and beverages.

## Figures and Tables

**Table 1:** Principal ingredients in beverage formulation [40,42].

Ingredient	Function in Beverage Formulation
Water	The major component of a beverage, constituting 85–98%. It has a carrier function for other ingredients and has significant effects on the taste, appearance and physical and microbiological stability of beverages during storage.
Carbohydrates	Give sweetness and texture to beverages; they have a synergistic role and give balance to flavorings. Sucrose, inverted sugar, glucose syrup and high-fructose corn syrup are the most common carbohydrates in beverages.
Sweeteners	Provide sweetness, reduce calories and have synergistic actions in beverages, improving their sensorial properties and stability. Aspartame, acesulfame K, sucralose and stevia are commonly used sweeteners.
Acidity regulators	Determine the sugar–acid balance, having an influence on the sensory properties. Citric acid and phosphoric acid are the most used acidity regulators.
Flavorings	Provide the final sensorycharacteristics (taste and smell). Solubility is the main parameter to be considered when using flavorings. Water-soluble, emulsion-based and spray-dried flavorings are the principal forms of flavoring used in beverages.
Colors	Make products more attractive to consumers, having an influence on the visual appearance of beverages.
Preservatives	Make products more attractive to consumers, influencing the visual appearance of beverages. Used to minimize microbial spoilage risk during storage. Potassium sorbate and sodium benzoate are the most commonly used preservatives in beverages.

**Table 2:** Fruit and vegetable waste used in functional beverages.

Type of Beverage	Waste Food Ingredient	Functionality	Reference
Apple–peach	Lemon peel (polyphenols)	Antioxidant and antimicrobial capacity increased during storage	[75]
Refreshing beverage	Sea buckthorn waste (phenolic compounds)	Antioxidant, antimicrobial and nutritional functionality	[81]
Tea	Pomegranate peel (flavonoids and tannins)	Increased antioxidant and bio-accessibility capacity of polyphenols	[76]
Energy drink	Melon seeds (flavonoids and phenolic acids)	DPPH radical inhibition, antimicrobial activity against *Staphylococcus aureus, E. coli, Bacillus cereus* and *Aspergillus niger*	[78]
Coffee type	Zalaca seeds (flavonoid compounds)	Antioxidant activity and diuretic effect on white male Wistar rats	[79]
Juice	Dragon fruit peel (polyphenol compounds)	Inhibition of free radicals	[77]
Iced tea	Cacao processing waste (phenolic compounds and dietary fiber)	Antioxidant activity and good nutritional properties	[82]
Juice	Orange and pomegranate peel (polyphenol compounds)	Antioxidant and antimicrobial activity increased during storage	[80]
Infusion	Grape pomace (phenolic acids and flavonoids)	Antioxidant and anti-inflammatory activity	[101]
Verjuice	Unripe grapes (organic acids and phenolic compounds)	Antimicrobial agent against *E. coli*, *L. monocytogenes*, *S. typhimurium* and *S. aureus*	[94]
Juice	Carrot pomace hydrolysate (β-carotene, polyphenol compounds)	Rich in polyphenolics, low sedimentation	[96]
Isotonic beverage	Lettuce, courgette, carrot, spinach (fiber, protein and minerals)	Good physicochemical properties	[75]
Juice	Cauliflower by-product powder (flavonoid compounds)	Antioxidant, enhances the nutritional value	[97]
Juice	Pumpkin (vitamin C, niacin and carotene)	High detoxification and antioxidant properties	[106]
Juice	Broccoli pomace (polyphenol content)	Antioxidant, higher content of soluble carbohydrates (lower fiber content) and proteins	[98]
Juice	Beetroot (phenolic and flavonoid compounds)	High amount of all minerals, enhances the beverage’s taste, flavor and antioxidant capacity	[99]
Juice	Sweet potato (anthocyanins, gallic acid, catechin, tryptophan)	High levels of bioactive compounds with antioxidant capacity	[108]
Juice	Eggplant peel (anthocyanins)	Antioxidant capacity against free radicals (ABTS and DPPH)	[102]
Juice	Prickly pear peels (pectic polysaccharides)	Antimicrobial activity during storage	[100]
Juice	Tomato waste (lycopene)	Antioxidant capacity against free radicals	[107]

**Table 3:** Functional beverage processing from dairy waste.

Type of Beverage	Waste Food Ingredient	Functionality	Reference
Fermented drink	Whey from Oaxaca cheese production	High bioavailability, nutritional value and antioxidant activity	[28]
Soursop whey beverage	Powder whey	High concentration of phenolic content; improvement in the antioxidant and antihypertensive activities; reduction in undesired minerals	[125]
Sport beverage	Liquid whey	Increase in protein and handgrip strength	[126]
Sport beverage	Ricotta cheese whey	Increase in volatile organic compounds; antimicrobial activity	[120]
Fruit beverage	Powder whey	Antioxidant activity, ACE (angiotensin-converting enzyme) inhibitory activity and α-glucosidase inhibition	[123]
Fermented milk beverage	Powder whey	Antioxidative, antibacterial, immune and cytomodulatory properties; ACE inhibition	[122]
Whey dairy beverages	Liquid whey	Inhibition activity on the viability of prostate cancer cells	[127]
Fermented probiotic beverage	Chhena cheese whey	Functional and nutritional qualities	[121]
Vegetable beverage	Liquid whey	Higher antioxidant activity and content of phenolic compounds, flavonoids and lipophilic pigments	[128]
Fermented beverage	Powder whey	Antioxidant and antimicrobial activity; ACE inhibitory activity	[124]

**Table 4:** Functional beverages with cereal waste as an ingredient.

Type of Beverage	Waste Food Ingredient	Functionality	Reference
Fermented beverage	Blue corn and black beans (phenolic compounds)	Antidiabetic and antihypertensive activity	[151]
Alcoholic beverage	Millet bran (vanillic, syringic, coumaric and ferulic acids)	Antioxidant activity related to the high polyphenol content	[156]
Fermented beverage	Quinoa flour (phenolic compounds)	Antioxidant activity, antihypertensive potential and sensorial acceptability	[157]
Juice	Sorghum Stalk (phenolic compounds)	Reduced oxidative stress and no changes in sensory properties	[149]
Fermented beverage	Malted barley (peptides)	Good physicochemical and antimicrobial properties	[154]
Multigrain beverage	Barnyard, foxtail kodo (phenolic compounds)	Prebiotic activity, low GI (glycemic index), high antioxidant activity in beverage	[148]
Fermented beverages	Rice bran (protein and dietary fiber)	Higher sensory acceptance, best shelf life and nutritional value	[155]
Tea	Corn tassel (phenolic compounds)	Antioxidant and high bioactivity	[150]
Multigrain probiotic beverage	Multigrain (oats, barley, buckwheat, rice) (phenolic compounds)	Antioxidant activity, excellent nutritional value, great stability	[153]

**Table 5:** Beverages obtained from fish waste.

Type of Beverage	Waste Food Ingredient	Functionality	Reference
Dairy beverage	Collagen (fish)	Increased nutritional properties, higher bioavailability and antioxidant capacity (ABTS)	[168]
Probiotic dairy beverage	Collagen (donated by industry)	Good physicochemical and microbiological parameters during storage	[47]
Fruit beverage	Collagen (fish)	Increase in collagen synthesis and improvement in protein folding	[176]
Sparkling water	Gelatin (skin, scales and fins)	Antibacterial properties and antioxidant activity against superoxide and the DPPH radical	[169]
Fruit juice	Hydrolysate collagen (cod skin)	Good nutritional and physicochemical properties	[165]
Apple juice and djulis extract	Collagen (green caviar)	Improved skin moisture and elasticity	[171]
Apple juice	Collagen (fish)	Improved skin hydration, brightness, texture	[175]
Non-alcoholic rose–apple beverage	Collagen (fish)	Antioxidant properties	[170]
Dairy beverage	Collagen (fish skin)	Good organoleptic, physicochemical and microbial properties	[172]
Omega-3 beverage	Collagen (donated by industry)	Improved wound healing rate	[174]
